# Supplementary material for: Two-Dimensional TeB Structures with Anisotropic Carrier Mobility and Tunable Bandgap
Source: Molecules. 2021 Oct 23;26(21):6404. doi: 10.3390/molecules26216404 (PMC8588529; doi:10.3390/molecules26216404)
Supplement: Supplementary file 1 [file molecules-26-06404-s001.zip › molecules-1425664-supplementary.pdf]

molecules

MDPI

Supporting Information

Two-dimensional TeB Structures with Anisotropic Carrier Mobility and Tunable Bandgap

Yukai Zhang [1], Xin Qu [1,2,*], Lihua Yang [1,*], Xin Zhong [1], Dandan Wang [1], Jian Wang [1], Baiyang Sun [1], Chang Liu [1], Jian Lv [2] and Jinghai Yang [1]

1 Key Laboratory of Functional Materials Physics and Chemistry of the Ministry of Education, Key Laboratory of Preparation and Application of Environmental Friendly Materials, College of Physics, Jilin Normal University, Changchun 130103, China; Lance19950815@163.com (Y.Z.); zhongxin19881119@163.com (X.Z.); mila880227@126.com (D.W.); wangjianphy@163.com (J.W.); ng68733384@163.com (B.S.); liuchang8081858@163.com (C.L.); jhyang1@jlnu.edu.cn (J.Y.)

2 International Center for Computational Method and Software, State Key Laboratory of Superhard Materials, Key Laboratory of Physics and Technology for Advanced Batteries (Ministry of Education), College of Physics, Jilin University, Changchun 130012, China; lvjian@jlu.edu.cn

* Correspondence: quxin515@163.com (X.Q.), yanglh@jlnu.edu.cn (L.Y.)

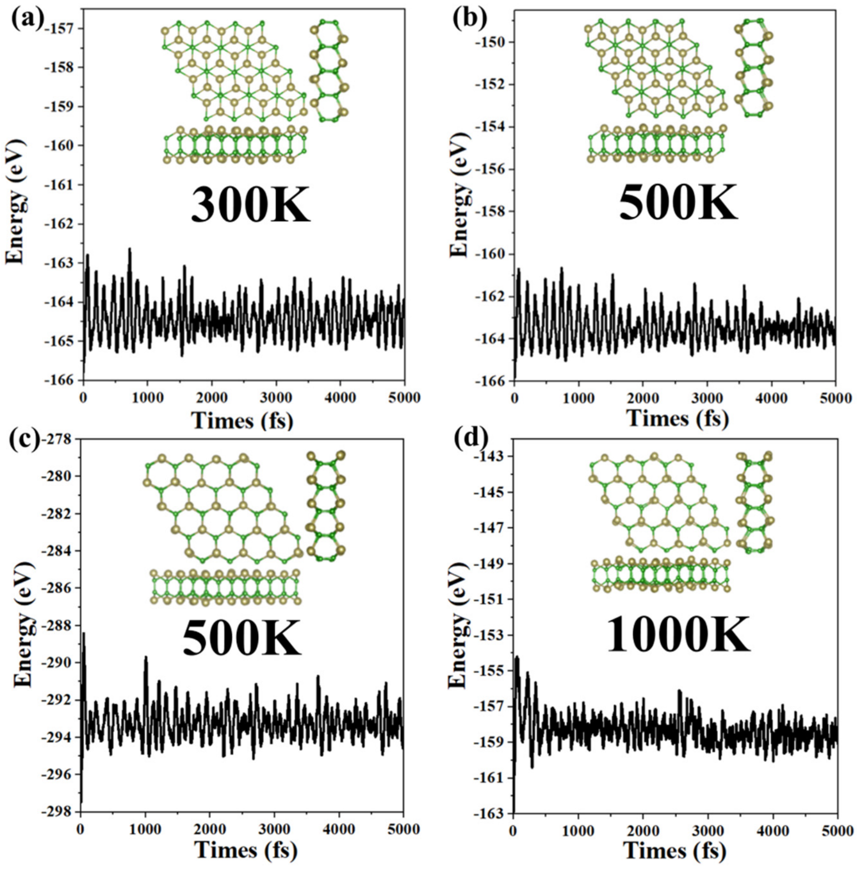

Figure S1. The energy fluctuation and the corresponding structure during the AIMD simulations at 300 K(**a**) and 500 K(**b**) for α-TeB, 500K(**c**) and 1000 K(**d**) for β-TeB, respectively.

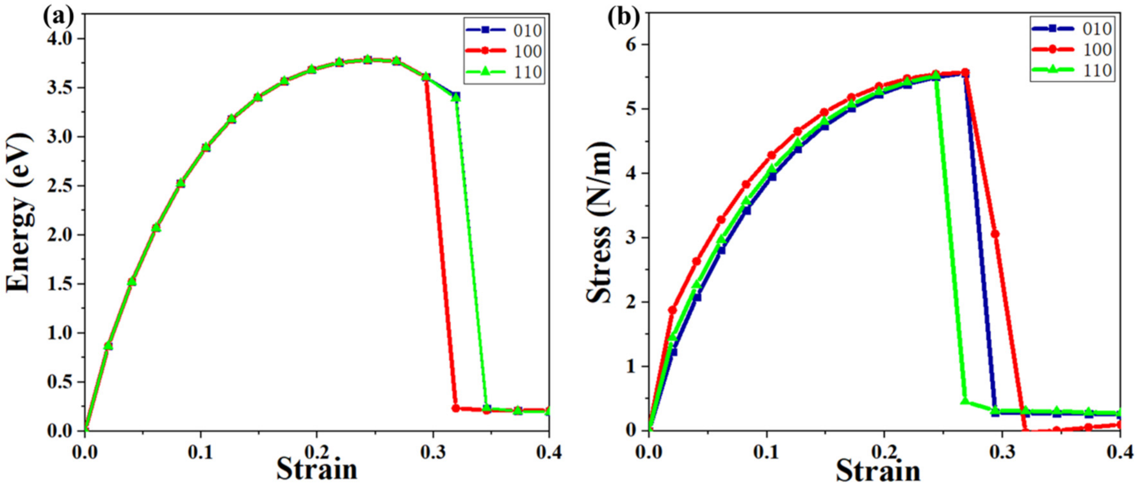

Figure S2. The ideal strength of α-TeB (**a**) and β-TeB (**b**) monolayers, respectively.

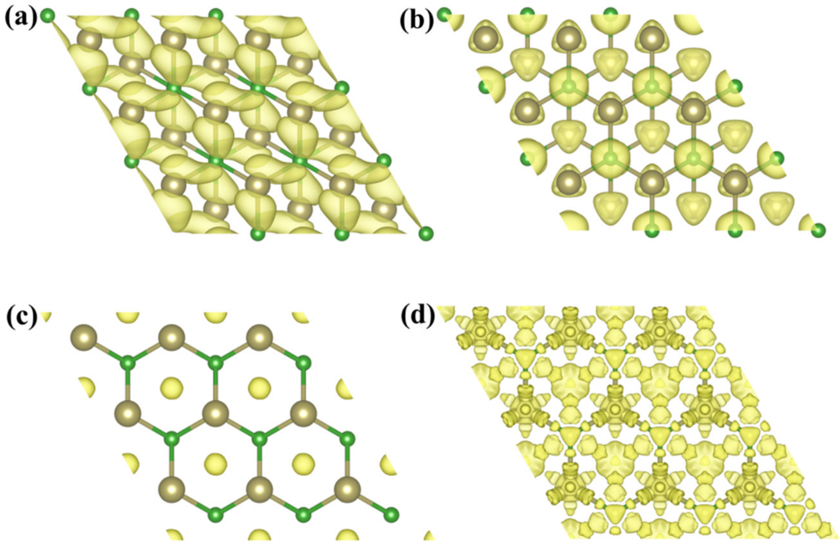

Figure S3. The band decomposed charge densities for VBM (**a**) and CBM (**b**) of α-TeB monlayer and The band decomposed charge densities for VBM (**c**) and CBM (**d**) of β-TeB monlayer.
